# Supplementary material for: An Engineered IFNγ-Antibody Fusion Protein with Improved Tumor-Homing Properties
Source: Pharmaceutics. 2023 Jan 22;15(2):377. doi: 10.3390/pharmaceutics15020377 (PMC9964878; doi:10.3390/pharmaceutics15020377)
Supplement: Supplementary file 1 [file pharmaceutics-15-00377-s001.zip › pharmaceutics-2141545-supplementary.pdf]

Supplementary Material

A **B**

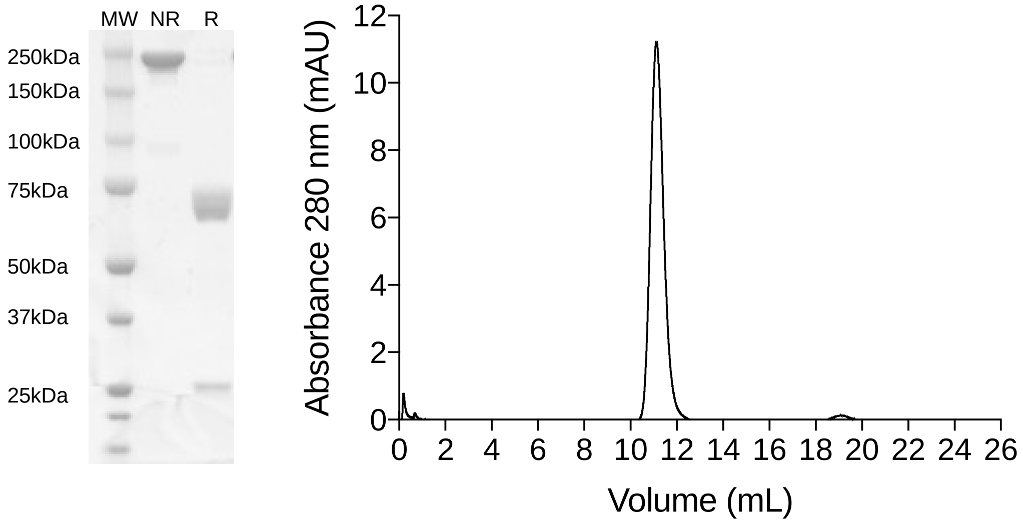

C

```
Light Chain:
EIVLTQSPGTLSLSPGERATLSCRASQSVSSSFLAWYQQKPGQAPRLLIYYASSRATGIPDRFSGSGSGTDFTLTIS
RLEPEDFAVYYCQQTGRIPPTFGQGTKVEIKRTVAAPSVFIFPPSDEQLKSGTASVVCLLNNFYPREAKVQWKVDNA
LQSGNSQESVTEQDSKDSTYSLSSTLTLSKADYEKHKVYACEVTHQGLSSPVTKSFNRGEC

Heavy Chain:
EVQLLESGGGLVQPGGSLRLSCAASGFTFSSFSMSWVRQAPGKGLEWVSSISGSSGTTYYADSV KGRFTISRDNSKN
TLYLQMNSLRAEDTAVYYCAKPFPYFDYWGQGTLVTVSSASTKGPSVFPLAPCSRSTSESTAAL GCLVKDYFPEPVT
VSWNSGALTSGVHTFPAVLQSSGLYSLSSVVTVPSSSLGTKTYTCNVDHKPSNTKVDKRVESKY GPPCPPCPAPEFL
GGPSVFLFPPKPKDTLMISRTPEVTCVVVDVSQEDPEVQFNWYVDGVEVHNAKTKPREEQFNST YRVVSVLTVLHQD
WLNGKEYKCKVSNKGLPSSIEKTISKAKGQPREPQVYTLPPSQEEMTKNQVSLTCLVKGFYPSD IAVEWESNGQPEN
NYKTTPPVLDSDGSFFLYSRLTVDKSRWQEGNVFSCSVMHEALHNHYTQKSLSLSLGGS QDPYVKEAENLKKYFNAG
HSDVADNGTLFLGILKNWKEESDRKIMQSQIVSFYFKLFKNFKDDQSIQKSVETIKEDMNVKFFNSNKKKRDDFEKL
TNYSVTDLNVQRKAIHELIQVMAELSPAAKT G
```

Figure S1 L19-IFNg TG biochemical characterization and amino acid sequence

(A) SDS-page, 10% gel in reducing (R) and non-reducing (NR) conditions. (**B)** Size exclusion chromatography profile. (**C**) Amino acid sequence.

A

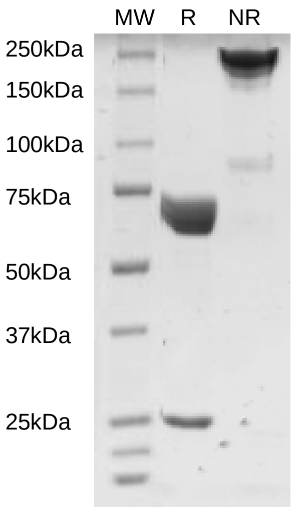

B

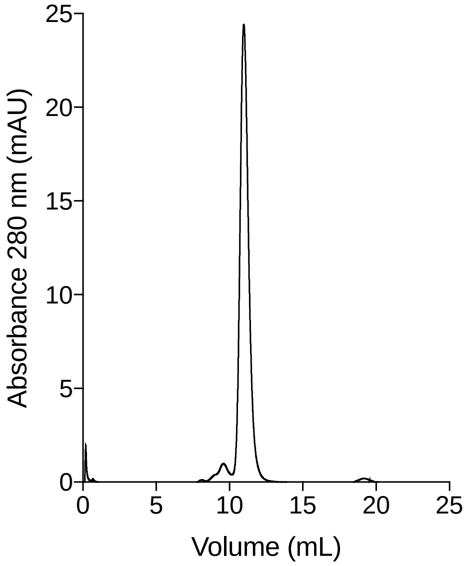

C

Light Chain:
EIVLTQSPGTLSLSPGERATLSCRASQSVSSSFLAWYQQKPGQAPRLLIYYASSRATGIPDRFSGSGSGTDFTLTIS
RLEPEDFAVYYCQQTGRIPPTFGQGTKVEIKRTVAAPSVFIFPPSDEQLKSGTASVVCLLNNFYPREAKVQWKVDNA
LQSGNSQESVTEQDSKDSTYSLSSTLTLSKADYEKHKVYACEVTHQGLSSPVTKSFNRGEC

Heavy Chain:
EVQLLESGGGLVQPGGSLRLSCAASGFTFSSFSMSWVRQAPGKGLEWVSSISGSSGTTYYADSV KGRFTISRDNSKN
TLYLQMNSLRAEDTAVYYCAKPFPYFDYWGQGTLVTVSSASTKGPSVFPLAPCSRSTSESTAAL GCLVKDYFPEPVT
VSWNSGALTSGVHTFPAVLQSSGLYSLSSVVTVPSSSLGTKTYTCNVDHKPSNTKVDKRVESKY GPPCPPCPAPEFL
GGPSVFLFPPKPKDTLMISRTPEVTCVVVDVSQEDPEVQFNWYVDGVEVHNAKTKPREEQFNST YRVVSVLTVLHQD
WLNGKEYKCKVSNKGLPSSIEKTISKAKGQPREPQVYTLPPSQEEMTKNQVSLTCLVKGFYPSD IAVEWESNGQPEN
NYKTTPPVLDSDGSFFLYSRLTVDKSRWQEGNVFSCSVMHEALHNHYTQKSLSLSLGGS QDPYVKEAENLKKYFNAG
HSDVADNGTLFLGILKNWKEESDRKIMQSQIVSFYFKLFKNFKDDQSIQKSVETIKEDMNVKFFNSNKKKRDDFEKL
TNYSVTDLNVQRKAIHELIQVMAELSPAAKT GKR

Figure S2 L19-IFNg KR biochemical characterization and amino acid sequence

(A) SDS-page, 10% gel in reducing (R) and non-reducing (NR) conditions. (**B)** Size exclusion chromatography profile. (**C**) Amino acid sequence.

```
Light Chain:
EIVLTQSPGTLSLSPGERATLSCRASQSVSSSFLAWYQQKPGQAPRLLIYYASSRATGIPDRFSGSGSGTDFTLTIS
RLEPEDFAVYYCQQTGRIPPTFGQGTKVEIKRTVAAPSVFIFPPSDEQLKSGTASVVCLLNNFYPREAKVQWKVDNA
LQSGNSQESVTEQDSKDSTYSLSSTLTLSKADYEKHKVYACEVTHQGLSSPVTKSFNRGEC

Heavy Chain:
EVQLLESGGGLVQPGGSLRLSCAASGFTFSSFSMSWVRQAPGKGLEWVSSISGSSGTTYYADSV KGRFTISRDNSKN
TLYLQMNSLRAEDTAVYYCAKPFPYFDYWGQGTLVTVSSASTKGPSVFPLAPCSRSTSESTAAL GCLVKDYFPEPVT
VSWNSGALTSGVHTFPAVLQSSGLYSLSSVVTVPSSSLGTKTYTCNVDHKPSNTKVDKRVESKY GPPCPPCPAPEFL
GGPSVFLFPPKPKDTLMISRTPEVTCVVVDVSQEDPEVQFNWYVDGVEVHNAKTKPREEQFNST YRVVSVLTVLHQD
WLNGKEYKCKVSNKGLPSSIEKTISKAKGQPREPQVYTLPPSQEEMTKNQVSLTCLVKGFYPSD IAVEWESNGQPEN
NYKTTPPVLDSDGSFFLYSRLTVDKSRWQEGNVFSCSVMHEALHNHYTQKSLSLSLGGS QDPYVKEAENLKKYFNAG
HSDVADNGTLFLGILKNWKEESDRKIMQSQIVSFYFKLFKNFKDDQSIQKSVETIKEDMNVKFFNSNKKKRDDFEKL
TNYSVTDLNVQRKAIHELIQVMAELSPAAKTGKRG
```

Figure S3 L19-IFNg KRG amino acid sequence

A

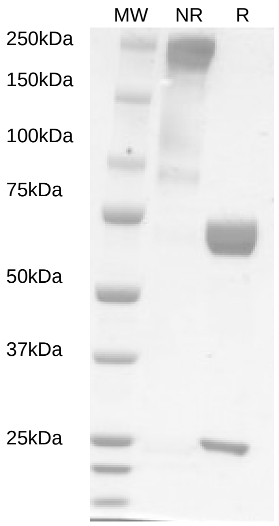

B

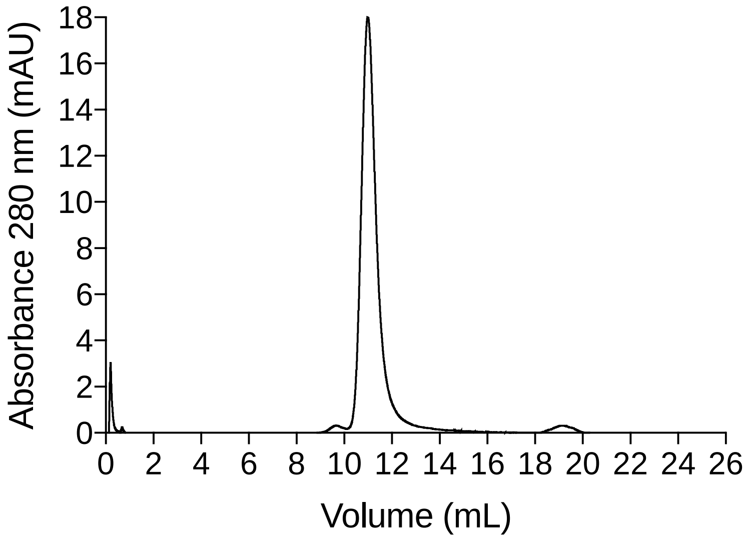

C

Light Chain:
EIVLTQSPGTLSLSPGERATLSCRASQSVSSSFLAWYQQKPGQAPRLLIYYASSRATGIPDRFSGSGSGTDFTLTIS
RLEPEDFAVYYCQQTGRIPPTFGQGTKVEIKRTVAAPSVFIFPPSDEQLKSGTASVVCLLNNFYPREAKVQWKVDNA
LQSGNSQESVTEQDSKDSTYSLSSTLTLSKADYEKHKVYACEVTHQGLSSPVTKSFNRGEC

Heavy Chain:
EVQLLESGGGLVQPGGSLRLSCAASGFTFSSFSMSWVRQAPGKGLEWVSSISGSSGTTYYADSV KGRFTISRDNSKN
TLYLQMNSLRAEDTAVYYCAKPFPYFDYWGQGTLVTVSSASTKGPSVFPLAPCSRSTSESTAAL GCLVKDYFPEPVT
VSWNSGALTSGVHTFPAVLQSSGLYSLSSVVTVPSSSLGTKTYTCNVDHKPSNTKVDKRVESKY GPPCPPCPAPEFL
GGPSVFLFPPKPKDTLMISRTPEVTCVVVDVSQEDPEVQFNWYVDGVEVHNAKTKPREEQFNST YRVVSVLTVLHQD
WLNGKEYKCKVSNKGLPSSIEKTISKAKGQPREPQVYTLPPSQEEMTKNQVSLTCLVKGFYPSD IAVEWESNGQPEN
NYKTTPPVLDSDGSFFLYSRLTVDKSRWQEGNVFSCSVMHEALHNHYTQKSLSLSLGGS QDPYVKEAENLKKYFNAG
HSDVADNGTLFLGILKNWKEESDRKIMQSQIVSFYFKLFKNFKDDQSIQKSVETIKEDMNVKFFNSNKKKRDDFEKL
TNYSVTDLNVQRKAIHELIQVMAELSPAAKT GKRKRSQMLFRG

Figure S4 L19-IFNg WT biochemical characterization and amino acid sequence

(A) SDS-page, 10% gel in reducing (R) and non-reducing (NR) conditions. (**B)** Size exclusion chromatography profile. (**C**) Amino acid sequence.

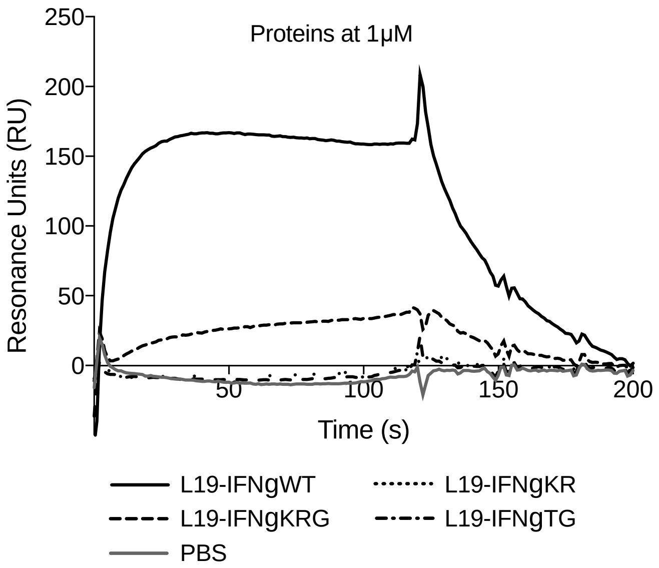

Figure S5 SPR analysis on IFNgR1 CM5 coated chip

Proteins at 1μM were analyzed on an IFNgR1 CM5 coated chip.

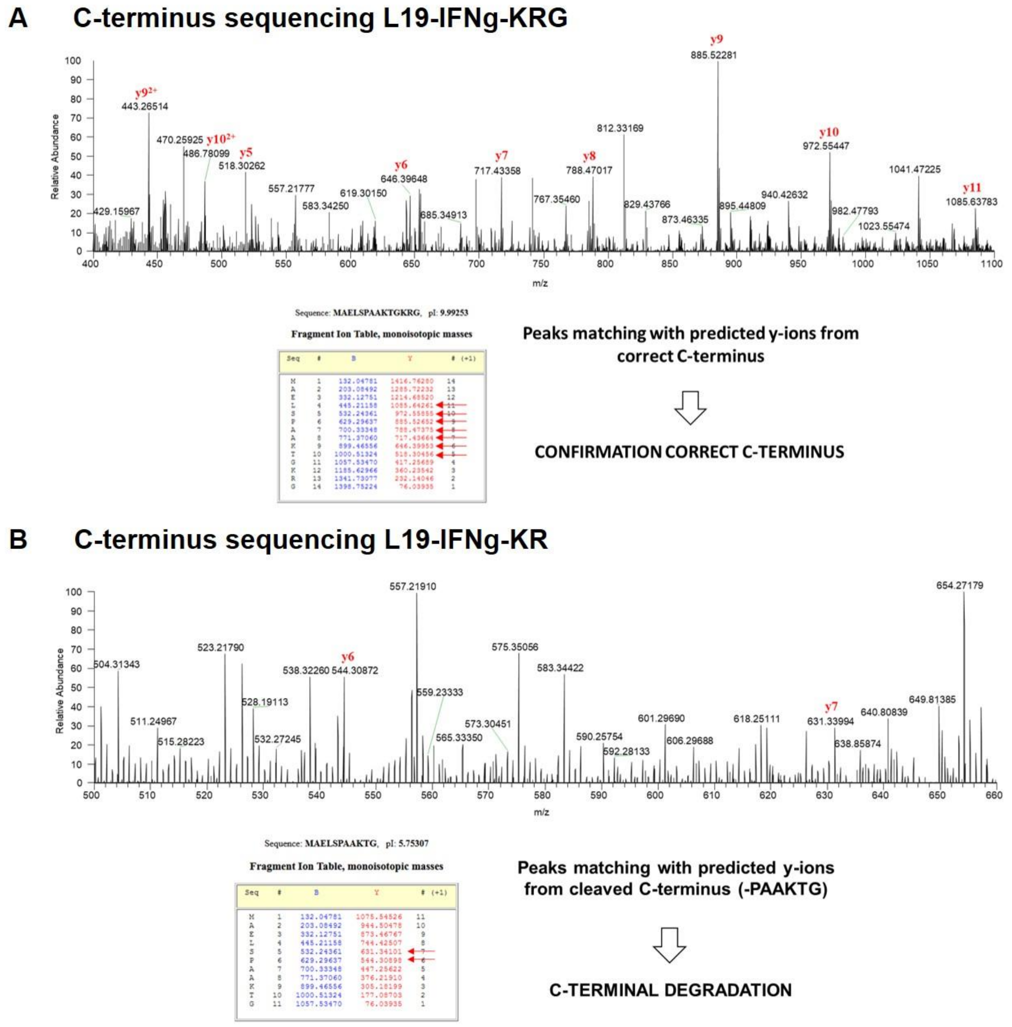

Figure S6 C-terminus sequence confirmation by Intact Mass Analysis

For L19-IFNg-KRG variant (**A**), several y-ions from the predicted fragmentation list were detected in the fragmentation spectrum, confirming its identity. For L19-IFNg-KR variant (**B**), no y-ions from the predicted fragmentation list were detected in the fragmentation spectrum. However, y-ions from the predicted fragmentation list of L19-IFNg-TG variant were identified indicating a C-terminal degradation.

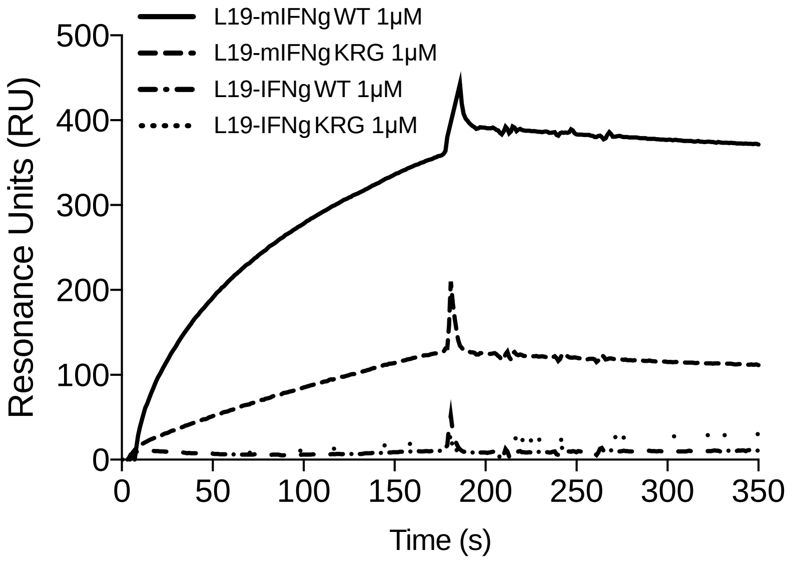

Figure S7 SPR analysis on mIFNgR1 CM5 coated chip

Proteins at 1μM were analyzed on a mIFNgR1 CM5 coated chip.

```
Light Chain:
EIVLTQSPGTLSLSPGERATLSCRASQSVSSSFLAWYQQKPGQAPRLLIYYASSRATGIPDRFSGSGSGTDFTLTIS
RLEPEDFAVYYCQQTGRIPPTFGQGTKVEIKRTVAAPSVFIFPPSDEQLKSGTASVVCLLNNFYPREAKVQWKVDNA
LQSGNSQESVTEQDSKDSTYSLSSTLTLSKADYEKHKVYACEVTHQGLSSPVTKSFNRGEC

Heavy Chain:
EVQLLESGGGLVQPGGSLRLSCAASGFTFSSFSMSWVRQAPGKGLEWVSSISGSSGTTYYADSV KGRFTISRDNSKN
TLYLQMNSLRAEDTAVYYCAKPFPYFDYWGQGTLVTVSSASTKGPSVFPLAPCSRSTSESTAAL GCLVKDYFPEPVT
VSWNSGALTSGVHTFPAVLQSSGLYSLSSVVTVPSSSLGTKTYTCNVDHKPSNTKVDKRVESKY GPPCPPCPAPEFL
GGPSVFLFPPKPKDTLMISRTPEVTCVVVDVSQEDPEVQFNWYVDGVEVHNAKTKPREEQFNST YRVVSVLTVLHQD
WLNGKEYKCKVSNKGLPSSIEKTISKAKGQPREPQVYTLPPSQEEMTKNQVSLTCLVKGFYPSD IAVEWESNGQPEN
NYKTTPPVLDSDGSFFLYSRLTVDKSRWQEGNVFSCSVMHEALHNHYTQKSLSLSLG GSGGGGSGGGSGGGGSGGGS
HGTVIESLESLNNYFNSSGIDVEEKSLFLDIWRNWQKDGDMKILQSQIISFYLRLFEVLKDNQAISNNISVIESHLI
TTFFSNSKAKKDAFMSIAKFEVNNPQVQRQAFNELIRVVHQLLPESSLRKRG
```

Figure S8 L19-mIFNg KRG amino acid sequence

A **B**

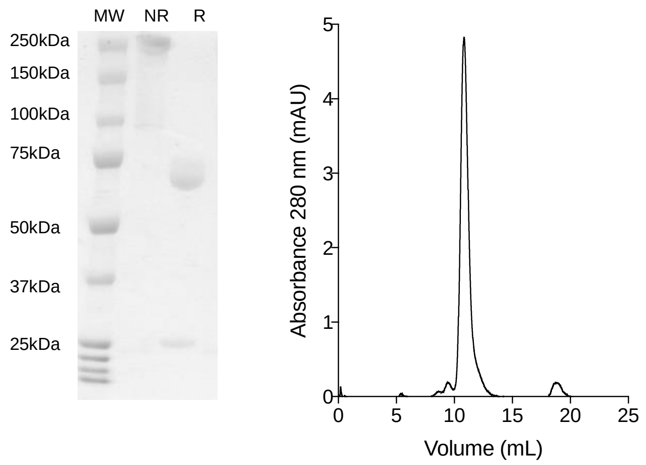

C

```
Light Chain:
EIVLTQSPGTLSLSPGERATLSCRASQSVSSSFLAWYQQKPGQAPRLLIYYASSRATGIPDRFSGSGSGTDFTLTIS
RLEPEDFAVYYCQQTGRIPPTFGQGTKVEIKRTVAAPSVFIFPPSDEQLKSGTASVVCLLNNFYPREAKVQWKVDNA
LQSGNSQESVTEQDSKDSTYSLSSTLTLSKADYEKHKVYACEVTHQGLSSPVTKSFNRGEC

Heavy Chain:
EVQLLESGGGLVQPGGSLRLSCAASGFTFSSFSMSWVRQAPGKGLEWVSSISGSSGTTYYADSV KGRFTISRDNSKN
TLYLQMNSLRAEDTAVYYCAKPFPYFDYWGQGTLVTVSSASTKGPSVFPLAPCSRSTSESTAAL GCLVKDYFPEPVT
VSWNSGALTSGVHTFPAVLQSSGLYSLSSVVTVPSSSLGTKTYTCNVDHKPSNTKVDKRVESKY GPPCPPCPAPEFL
GGPSVFLFPPKPKDTLMISRTPEVTCVVVDVSQEDPEVQFNWYVDGVEVHNAKTKPREEQFNST YRVVSVLTVLHQD
WLNGKEYKCKVSNKGLPSSIEKTISKAKGQPREPQVYTLPPSQEEMTKNQVSLTCLVKGFYPSD IAVEWESNGQPEN
NYKTTPPVLDSDGSFFLYSRLTVDKSRWQEGNVFSCSVMHEALHNHYTQKSLSLSLG GSGGGGSGGGSGGGGSGGGS
HGTVIESLESLNNYFNSSGIDVEEKSLFLDIWRNWQKDGDMKILQSQIISFYLRLFEVLKDNQAISNNISVIESHLI
TTFFSNSKAKKDAFMSIAKFEVNNPQVQRQAFNELIRVVHQLLPESSLRKRKRSRS
```

D

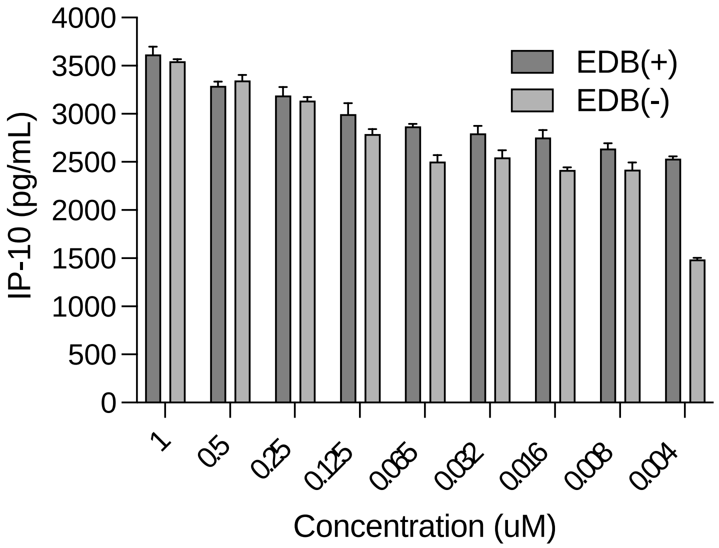

Figure S9 L19-mIFNg WT biochemical characterization and amino acid sequence

(A) SDS-page, 10% gel in reducing (R) and non-reducing (NR) conditions. (**B)** Size exclusion chromatography profile. (**C**) Amino acid sequence. (D) IP-10

release on TIB-49 cells exposed to titration of L19-mIFNg KRG in coated EDB(+) and non-coated EDB(-) wells.

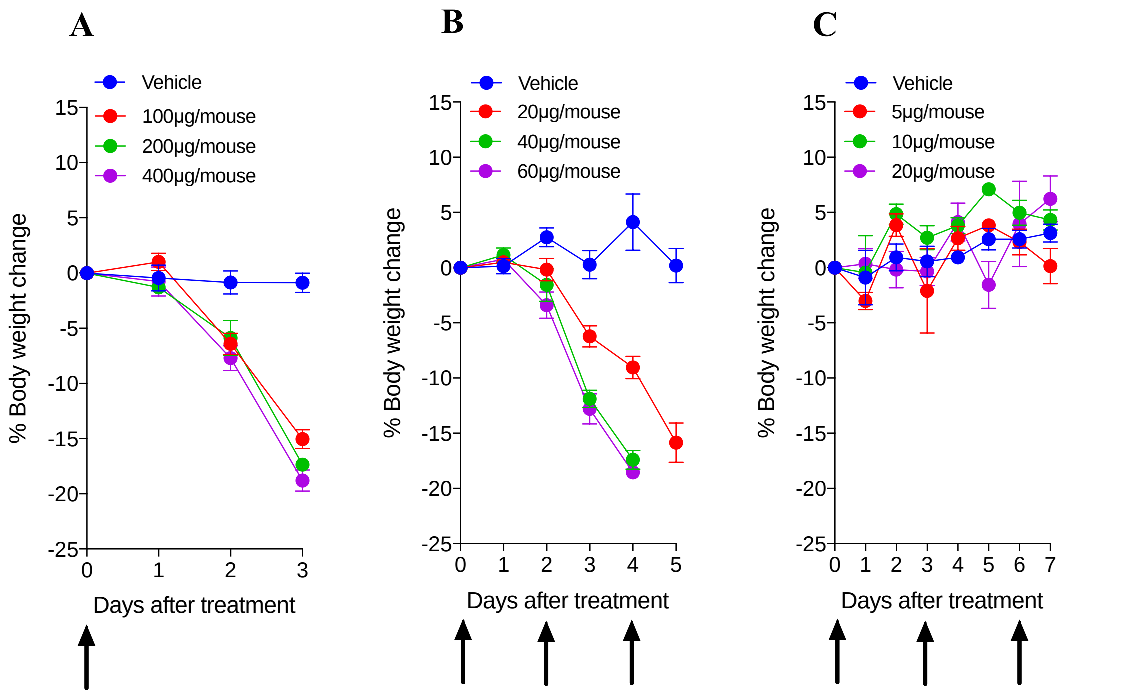

Figure S10 Dose escalation study

WEHI-164 tumor bearing mice intravenously injected at the following dose and schedules: (**A**) 400µg, 200µg, and 100µg with a single injection. (**B**) 60µg, 40µg and 20µg every 48h. (**C**) 20µg, 10µg and 5µg every 72h interval. Body weight loss equal or superior to 15% respect to the average body weight was considered as an endpoint. Data represent mean % weight (± SEM), n = 4 mice per group.

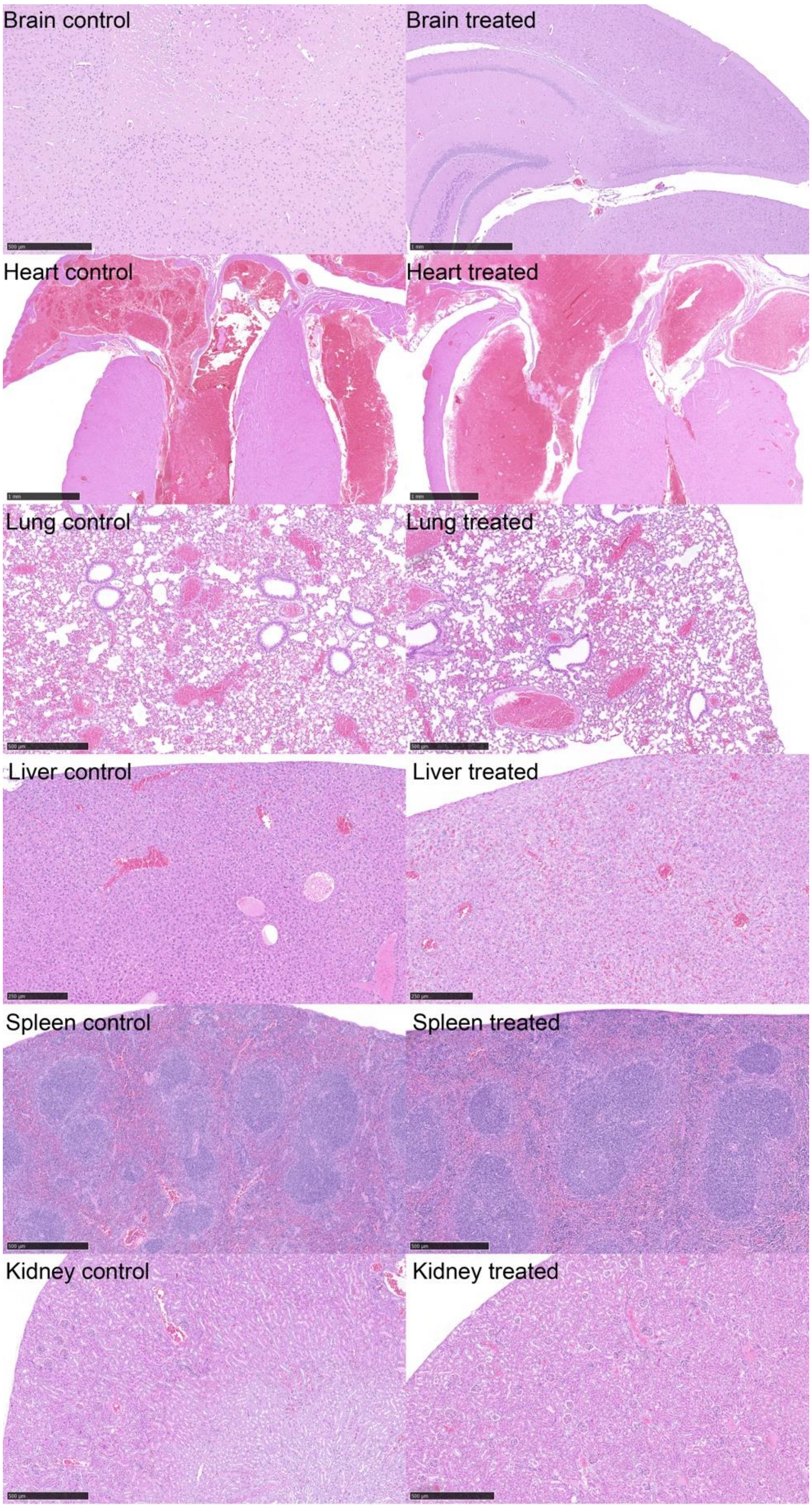

Figure S11 Histopathological analysis on tumor bearing mice

H&E staining of organs after L19-mIFNg KRG (right panels) treatment compared to Saline (left panels).

A **B**

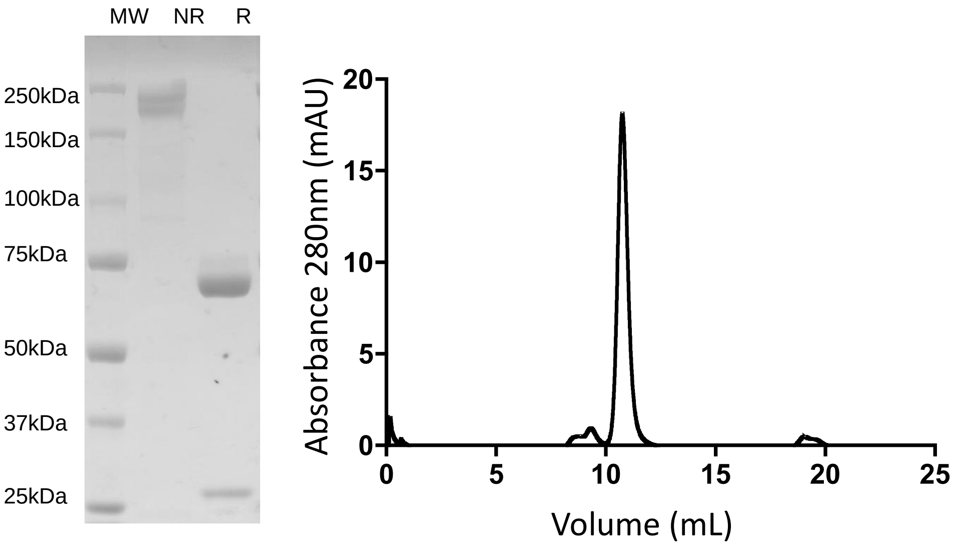

C

```
Light Chain:
SSELTQDPAVSVALGQTVRITCQGDSLRSYYASWYQQKPGQAPVLVIYGKNNRPSGIPDRFSGSSSGNTASLTITGA
QAEDEADYYCQSSPLNRLAVVFGGGTKLTVLGQPKAAPSVTLFPPSSEELQANKATLVCLISDFYPGAVTVAWKADS
SPVKAGVETTTPSKQSNNKYAASSYLSLTPEQWKSHKSYSCQVTHEGSTVEKTVAPTECS

Heavy Chain:
EVQLLESGGGLVQPGGSLRLSCAASGFTFSSYAMSWVRQAPGKGLEWVSAISGSGGSTYYADSV KGRFTISRDNSKN
TLYLQMNSLRAEDTAVYYCAKSPKVSLFDYWGQGTLVTVSSASTKGPSVFPLAPCSRSTSESTA ALGCLVKDYFPEP
VTVSWNSGALTSGVHTFPAVLQSSGLYSLSSVVTVPSSSLGTKTYTCNVDHKPSNTKVDKRVES KYGPPCPPCPAPE
FLGGPSVFLFPPKPKDTLMISRTPEVTCVVVDVSQEDPEVQFNWYVDGVEVHNAKTKPREEQFN STYRVVSVLTVLH
QDWLNGKEYKCKVSNKGLPSSIEKTISKAKGQPREPQVYTLPPSQEEMTKNQVSLTCLVKGFYP SDIAVEWESNGQP
ENNYKTTPPVLDSDGSFFLYSRLTVDKSRWQEGNVFSCSVMHEALHNHYTQKSLSLSLGGSGGG GSGGGSGGGGSGG
GSHGTVIESLESLNNYFNSSGIDVEEKSLFLDIWRNWQKDGDMKILQSQIISFYLRLFEVLKDN QAISNNISVIESH
LITTFFSNSKAKKDAFMSIAKFEVNNPQVQRQAFNELIRVVHQLLPESSLRKRG
```

Figure S12 KSF-mIFNg KRG biochemical characterization and amino acid sequence

(A) SDS-page, 10% gel in reducing (R) and non-reducing (NR) conditions. (**B)** Size exclusion chromatography profile. (**C**) Amino acid sequence.

Original SDS-Page images

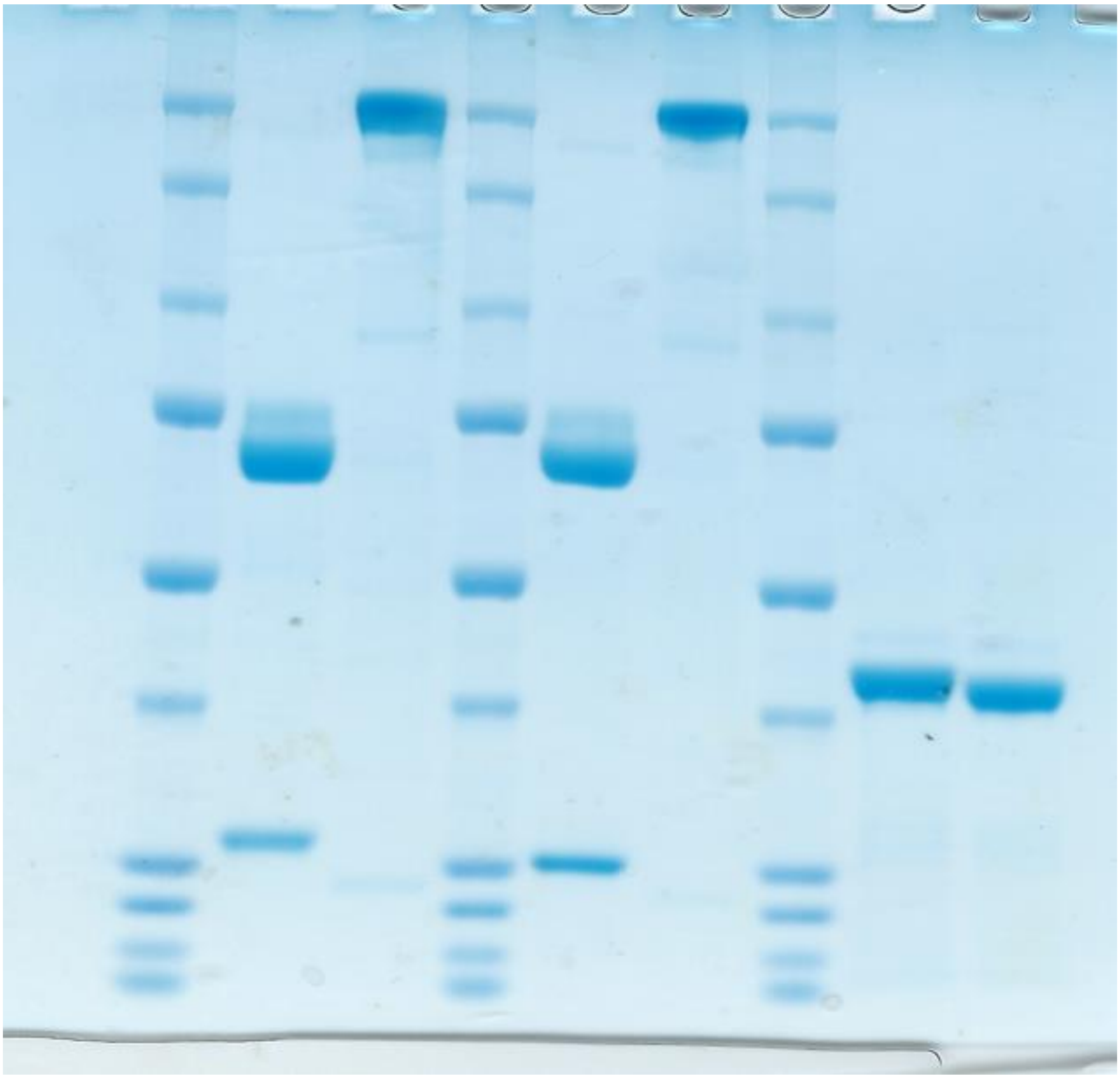

Original SDS-Page from Figure 1. Lanes description from left to right, in **bold** the figure present in the manuscript: molecular weight ladder / Protein reducing condition / Protein non-reducing condition / molecular weight ladder / **L19-IFNg KRG Figure 1 reducing condition / L19-IFNg KRG Figure 1 non-reducing condition** / molecular weight ladder / Protein reducing condition / Protein non-reducing condition

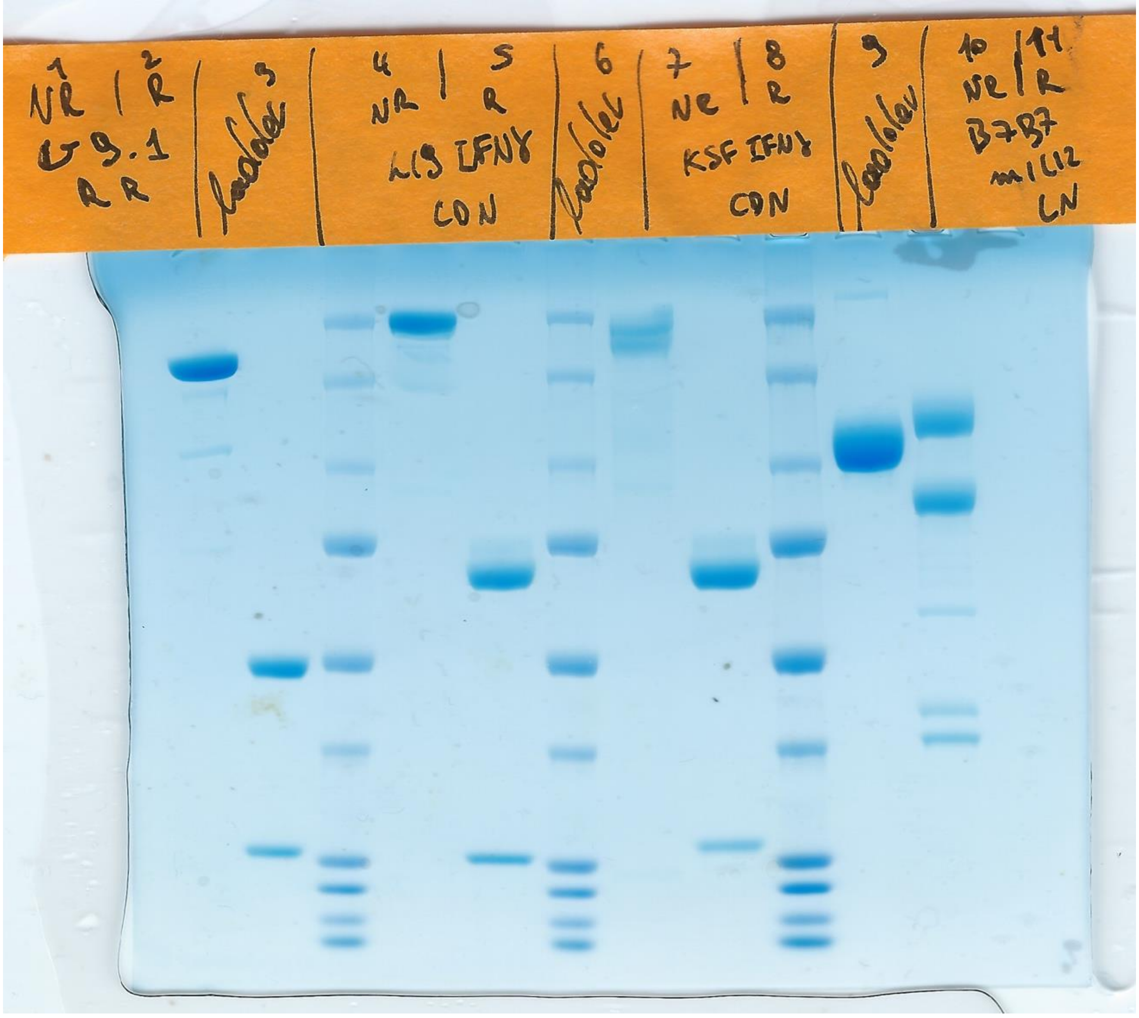

Original SDS-Page from Figure 3. lanes description from left to right, in **bold** the figure present in the manuscript: Protein non-reducing condition / Protein reducing condition / molecular weight ladder / **L19-mIFNg KRG Figure 3 non-reducing condition / L19-mIFNg KRG Figure 3 reducing condition** / molecular weight ladder / Protein non-reducing condition / Protein reducing condition / molecular weight ladder / Protein non-reducing condition / Protein reducing condition
